# Navigating the Challenges in Setting Up a Sustainable Open-Heart Surgery Unit in a Resource-Constrained Environment in Northern Nigeria: Model and Strategies

**DOI:** 10.21470/1678-9741-2023-0107

**Published:** 2024-07-17

**Authors:** Ikechukwuka Ifeanyichukwu Alioke, Francis Luke Idoko, Olugbenga Olusola Abiodun, Ogechi Chinagosi Daisy Maduka, Emmanuel Ozoemena Ugwu, Tina Anya, Salau Ibrahim Layi, Oc Nzewi

**Affiliations:** 1 Cardiothoracic Surgery Unit, Federal Medical Centre, Abuja, Nigeria; 2 Adult Cardiology Unit, Federal Medical Centre, Abuja, Nigeria.; 3 Paediatric Cardiology Unit, Federal Medical Centre, Abuja, Nigeria.; 4 Department of Anaesthesia, Federal Medical Centre, Abuja, Nigeria.; 5 Cardiac Surgeon, Save A Heart Foundation, United Kingdom.

**Keywords:** Cardiopulmonary Bypass, Nigeria, Cardiac Surgical Procedures, Government, Strategies, Self-Sustainable

## Abstract

**Introduction:**

Cardiac surgery requiring cardiopulmonary bypass had been unavailable in Northern Nigeria and the federal capital territory of Nigeria regularly. Several attempts in the past at setting up this service in a self-sustaining manner in Northern Nigeria had failed. This paper is a contrasting response to an earlier publication that emphasized the less-than-desirable role played by international cardiac surgery missions in the evolution of a sustainable open-heart surgery program in Nigeria.

**Methods:**

The cardiothoracic unit of Federal Medical Centre, Abuja, was established on March 1, 2021, but could not conduct safe open-heart surgery. The model and strategies employed in commencing open-heart surgeries, including the choice of personnel training within the country and focused collaboration with foreign missions, are discussed. We also report the first seven patients to undergo cardiac surgery under cardiopulmonary bypass in our government-run hospital as well as the transition from foreign missions to local team operations.

**Results:**

Seven patients were operated on within the first six months of setting up with high levels of skill transfer and local team participation, culminating in one of the operations entirely carried out by the local team of personnel. All outcomes were good at an average of one-year follow-up.

**Conclusion:**

In resource-constrained government-run hospitals, a functional, safe cardiac surgery unit can be set up by implementing well-planned strategies to mitigate encountered peculiar challenges. Furthermore, with properly harnessed foreign missions, a prior-trained local team of personnel can achieve independence and become a self-sustaining cardiac surgery unit within the shortest possible time.

## INTRODUCTION

For this article, we define open-heart surgery as a surgical procedure that requires an incision into the heart, thus exposing one or more of the cardiac chambers, or requires the institution of a cardiopulmonary bypass^[1]^.There has been a dearth of regular open-heart surgery in Northern Nigeria^[2]^. Few facilities that attempted such rescinded due to the high cost of their establishment and sustenance. The cardiothoracic unit of Federal Medical Centre, Abuja, was established on March 1, 2021, with the appointment of a substantive full-time cardiothoracic surgeon.

The thoracic and vascular surgical practice was commenced immediately. With a list of over 60 patients with surgical heart diseases seen on an out-patient basis, it became necessary for the unit to embark on incorporating open-heart surgery in the list of the services rendered, culminating in the first open-heart surgery missions in collaboration with Save A Heart Foundation, United Kingdom, (a foreign cardiac surgery missions team) in February 2022, within the first year of the unit’s existence.

The strategies put in place (in terms of personnel training, choice of equipment procured and installed, lobbying for management support, and choice of collaborating partners), as well as the challenges faced and surmounted towards setting up an indigenous, self-sustainable cardiac surgical program in a government-run hospital, are discussed below.

## METHODS

**Fig. 1:**
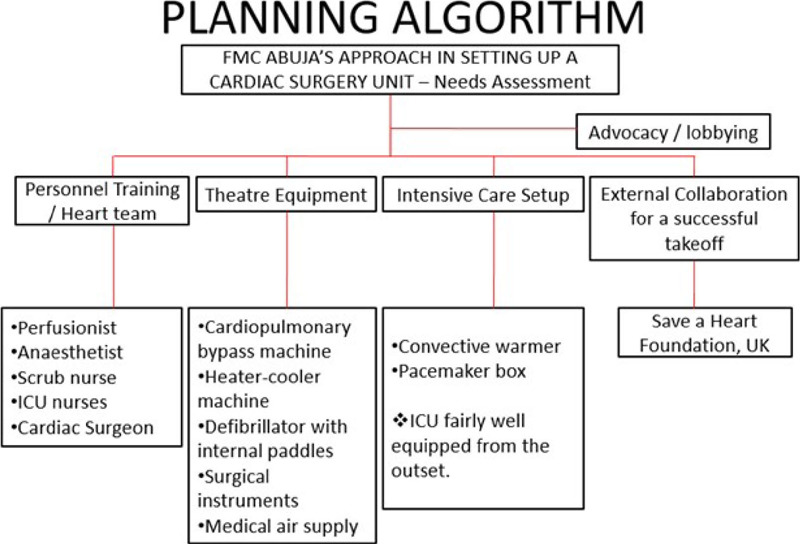
Planning algorithm for commencement of open-heart surgery at Federal Medical Centre (FMC), Abuja, Nigeria. ICU=intensive care unit; UK=United Kingdom

Considerable effort was made to minimize the cost of setting up a cardiac surgery centre without compromising minimum standards. To achieve the aforementioned, the following strategies were employed (Figure 1): advising the management on suppliers of equipment with competitive prices; procurement of some used equipment in very good working condition to minimize cost while achieving the desired result (cardiopulmonary bypass machine, dual-chamber heater-cooler machine, convective warmer, pacemaker box); initial training of personnel at a local centre with one of the highest volume of cardiac surgeries (Tristate Cardiovascular Centre, Babcock University Teaching Hospital, Ogun State); procurement of equipment in decreasing order of cost; reaching out to Save A Heart Foundation who fixed an early date, constraining the management at a time when “financial fatigue” was setting in; and encouraging adequate media publicity after the missions (the management has to “win” if our cardiac surgery program is to receive the much-desired support).

The first phase of planning entailed securing the minimum for successful adult cardiac surgery missions and giving the management the desired media publicity. To achieve this, the following action points were carried out, viz: procurement of the minimum required equipment, personnel training, and continuous advocacy and lobbying with the hospital management; also, a pioneer open-heart surgery mission was organized to test-run the system.

The second phase entailed planning for a sustainable cardiac surgery program. Following the pioneer open-heart surgery missions, areas of deficiencies were identified and addressed to set the course for a sustainable program. More needed equipment was procured. More personnel were encouraged to go for further training. More open-heart surgery missions were organized with an understanding to permit the full participation of local heart team members under close supervision by the visiting counterparts while progressively scaling down on the number of visiting participants in subsequent missions as the local team members developed competence and confidence. Furthermore, patients were scheduled for operation by the local heart team in between missions, under the supervision of selected more experienced visiting personnel, to further boost the confidence of the local team. An accessible, acceptable, and affordable source of consumable supplies was sought and secured. The hospital management was engaged to allow for some partial autonomy for the unit to reduce the bureaucratic delays involved in procurements. More partnerships and sponsors of cardiac surgery for indigent patients were sought, along with continued advocacy in support of the program.

## RESULTS

Contrary to the experience of some authors with the role of foreign cardiac surgery missions in the development of a sustainable open-heart surgery program^[3]^, we sought collaboration with Save A Heart Foundation, United Kingdom, with clearly-defined terms captured in a memorandum of understanding. The terms were primarily geared towards the transfer of competence and guidance in order to develop a self-sustaining program within a stipulated time frame, as opposed to a “surgical safari” as mentioned in a previous publication by Nwafor et al.^[3]^. There was a plan for periodic evaluation of the progress made in achieving the abovementioned aim. The specific role played by the foreign mission team included a physical assessment of the setup for open-heart surgery in our hospital, recommendations for improvement of setup, and planning for the first open-heart surgery mission. The specific objectives of the mission were to test the capacity of our hospital to carry out safe open-heart surgery, to introduce our local heart team to the rigours of cardiac evaluation and perioperative care, and to create an opportunity for transfer of skills and competencies towards establishing an indigenous, sustainable open-heart surgery program.

During the missions, four patients were safely operated on and discharged. The ability of the local heart team to work in synergy and coordination was tested. The local heart team members enjoyed an unparalleled skill transfer for first-ever missions in a new setup. Areas of inadequacy were identified and addressed.

Following the missions with Save A Heart Foundation, three more open-heart surgeries have been carried out in the subsequent three months, with the local heart team as the primary team operating on one of the patients (mechanical mitral valve replacement) (Table 1).

**Table 1:** Summary of patients operated on.

Serial order of patients	Age (years)	Gender	Diagnosis	EuroSCORE II	Operation done	Follow-up duration	Outcome
1	28	Female	Mitral stenosis	0.96	Mechanical mitral valve replacement	13 months	Alive and well
2	39	Female	Mitral regurgitation	0.82	Mechanical mitral valve replacement	13 months	Alive and well
3	65	Female	Coronary artery disease	1.83	Coronary artery bypass grafting	13 months	Alive and well
4	45	Female	Mitral and tricuspid regurgitation	0.83	Mechanical mitral valve replacement with De Vega tricuspid suture annuloplasty	13 months	Alive and well
5	42	Female	Mitral stenosis	0.69	Mechanical mitral valve replacement	10 months	Alive and well
6	5	Male	Atrial septal defect	@@	Patch closure	9 months	Alive and well
7	4	Male	Atrial septal defect	@@	Patch closure	9 months	Alive and well

EuroSCORE=European System for Cardiac Operative Risk Evaluation

## DISCUSSION

Setting up a sustainable cardiac surgery centre is capital intensive and requires highly specialized and skilled personnel for optimal patient safety^[4]^. These luxuries aren’t readily available in a relatively new government-run health institution in Nigeria. Optimal training of cardiac surgery personnel for Nigerians had largely been held outside our sub-region, leading to a higher cost of personnel training. This is due to the relatively lower volume of cardiac surgery activity in individual centres in Nigeria compared to the more established centres outside our sub-region^[5,6]^. Our guiding principle in the strategies employed in setting up a safe open-heart surgery program in our institution was to employ cost-effective measures that’ll yield acceptable results. In other centres, cardiac surgery units were usually set up as independent or semi-independent units within a hospital. Due to the dearth of facilities and space, the program, in this initial phase, was incorporated into the already-existing structure rendering surgical services in the hospital. The already existing structure had four standard theatre suites, one of which was large enough to accommodate the equipment needed for open-heart surgery, a well-equipped 10-bedded intensive care unit (ICU), and the surgical wards. Furthermore, a list of the equipment needed was itemized after evaluating the inventory of equipment already in existence in the hospital. Used equipment (in very good working condition) were bought from the United Kingdom and United States of America rather than the brand-new versions. For personnel training, select members of staff were sponsored to train in a local centre with the highest volume of cardiac surgical practice^[6]^. These measures instituted above greatly reduced the initial cost of setting up.

Contrary to the belief and experience of some authors^[3]^, the role of visiting mission organizations in our successful takeoff and transition to self-sustainability cannot be overemphasized. In our experience, a well-thought-out engagement with visiting mission teams, as well as a constant review of the progress being made towards the achievement of the overall aim of the partnership, will help in achieving an indigenous, self-sustainable program in the shortest time possible. Our progress in partnership with Save A Heart Foundation is arguably the fastest in Nigeria in establishing an indigenous program, with seven open-heart surgeries taking place in our institution^[6]^ within three months of commencement, one of which was performed by the local team of personnel. Other authors have also reported the positive role of visiting mission teams in the evolution of their open-heart surgery programs^[7]^.

**Fig. 2:**
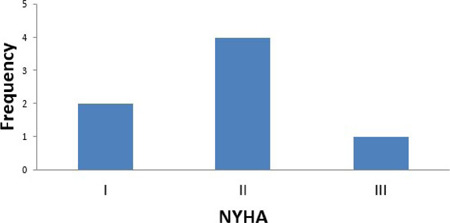
New York Heart Association (NYHA) classification of patients.

Being a new centre for cardiac surgery in Northern Nigeria, our team were particularly interested in good outcomes to win the confidence of those seeking similar services in the South and abroad as well as to boost the confidence of the local team members. Careful patient selection and meticulous preoperative optimization were key in ensuring good outcomes. This is evidenced by the good New York Heart Association and European System for Cardiac Operative Risk Evaluation scores of the patients (Figure 2). The complexity of cases undertaken will increase as the unit becomes more established.

**Fig. 3:**
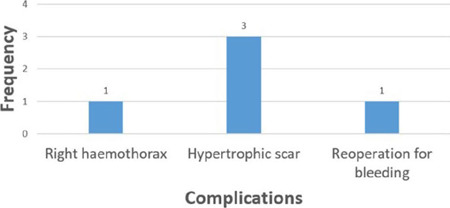
Complications of operation.

**Fig. 4:**
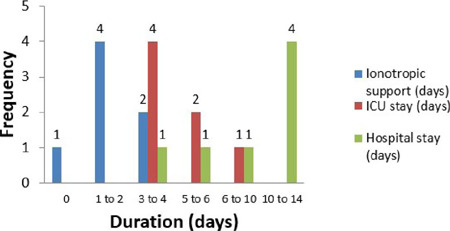
Duration of ionotropic support, intensive care unit (ICU) stay, and hospital stay. The mean ionotropic support was 2 days. The mean ICU stay was 4.8 days. The mean length of postoperative stay was 9.4 days.

**Fig. 5:**
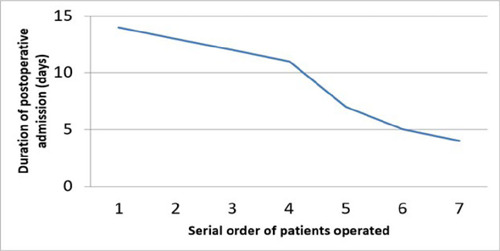
Trend of the duration of postoperative hospital admission.

The complications (Figure 3) encountered, mean duration of ionotropic support, ICU admission (Figure 4), postoperative hospital stay (Figure 5), and complications encountered were not out of the ordinary. When we commenced operations, we relied on protocols from centres in the United Kingdom. However, as we progressed, we deemed it necessary to modify and improve on some of the protocols within acceptable limits and clinical recommendations. Hence the progressive reduction in the duration of postoperative admission of the patients. One such significant modification was the commencement of warfarin at 10 mg daily for those having mechanical valves implanted, with subsequent downward adjustment as indicated. This enabled the patients to achieve therapeutic levels of anticoagulation faster with consequent earlier discharge. Earlier in the program, we usually commenced at 5 mg and increased as indicated which sometimes delayed discharge.

**Fig. 6:**
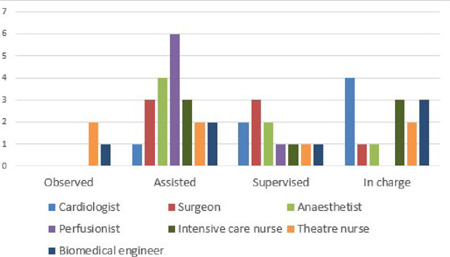
Overall participation of local heart team members.

Suffice it to note that excellent skills transfer was the goal of the foreign cardiac missions by Save A Heart Foundation, United Kingdom, as evidenced by the level of participation of the local heart team members (Figure 6). Little wonder we were able to perform a mitral valve replacement independently.

A high volume of open-heart surgeries performed in our institution is needed to maintain the skills of our trained personnel and help sustain the program. There is a lack of insurance coverage for open-heart surgery in Nigeria. Treatment of congenital abnormalities requiring advanced surgical procedures, *e.g.*, tetralogy of Fallot, atrial septal defect, ventricular septal defect, and by extension, all open-heart surgeries, was listed in the total exclusion list of procedures not covered by the Nigerian National Health Insurance Scheme^[8]^. Where patients can afford the out-of-pocket payment, there’s either lack of awareness of the availability of such services within the country or a lack of confidence in the capability of the local teams in the country. Although our institution is the only government-run hospital offering fairly routine open-heart surgery in the whole of Northern Nigeria, these abovementioned factors have hindered the much-desired increase in the volume of surgeries being performed. To mitigate this, widespread media publicity of the success rates of our open-heart surgeries was carried out. This has helped to build up our patient referrals. To finance the cost of surgeries for indigent patients, a Patients’ Indigent Fund exists in our institution. Furthermore, sponsorships and partnerships with local and foreign organizations are being sought for and utilized to finance/subsidize open-heart surgeries for such indigent patients. However, incorporating open-heart surgery into the list of procedures covered by the National Health Insurance Scheme will help change the narrative.

**Fig. 7:**
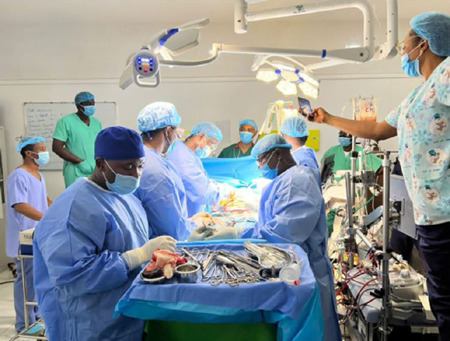
The local heart team replacing a diseased mitral valve.

**Fig. 8:**
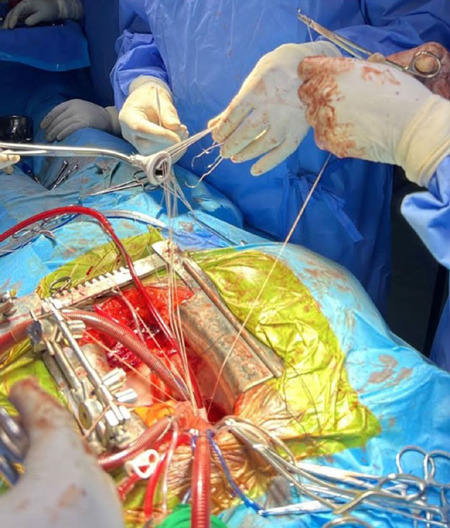
An On-X® mitral valve is sutured in place by the local heart team.

**Fig. 9:**
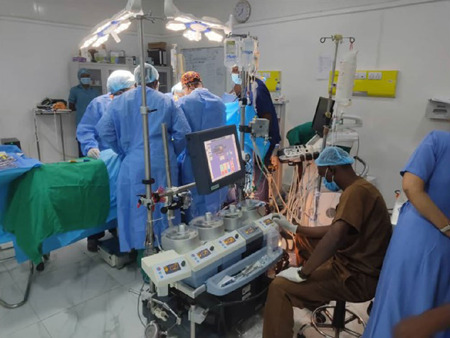
Perfusionist operating the heart-lung machine.

Other modalities being instituted to ensure sustainability include enabling every member of the heart team to own the program, as well as institute plans for additional remuneration of the local heart team members to compensate for the additional work being done, as open-heart surgery is labour-intensive^[9]^. Pictures of our setup during operations are captured in Figures 7, 8 and 9.

### Limitations

The lack of insurance cover for cardiac surgery has limited the number of cases done and slowed down the pace of transition to an indigenous practice.

## CONCLUSION

The approach employed in setting up open-heart surgical practice in the Federal Medical Centre, Abuja, may not be the most ideal. However, it was considered most suitable for achieving our goal within a relatively short period. In resource-constrained government-run hospitals, a functional, safe cardiac surgery unit can be set up by implementing well-planned strategies to mitigate encountered peculiar challenges. Furthermore, with properly harnessed foreign missions, a prior-trained local team of personnel can achieve complete independence and become a self-sustaining cardiac surgery unit within the shortest possible time.

## Abbreviations, Acronyms & Symbols

EuroSCORE: European System for Cardiac Operative Risk Evaluation
ICU: Intensive care unit
NYHA: New York Heart Association
UK: United Kingdom
